# Therapy group following a Ketamine treatment – a case report of a patient with resistant depression

**DOI:** 10.1192/j.eurpsy.2022.1442

**Published:** 2022-09-01

**Authors:** H. Yaniv, V. Savlev

**Affiliations:** Kfar Shaul Hospital, Closed Unit, Jerusalem, Israel

## Abstract

**Introduction:**

There are some patients that remain resistant to treatment for major depressive disorder - who were treated with two or more different medications, and did not demonstrate any improvement in their mental-state. These patients can be treated with a new treatment – Esketamine. The recommended Esketamine treatment protocol includes 8-treatment sessions, each session lasts about two hours. In our clinic, we added a therapy group after each treatment. The therapy group is led by two co-therapist and lasts 30 minutes. The patients are invited to share their experiences from the session.

**Objectives:**

We will present a case report of a 44 year old man, that suffers from a major-depression for years, with symptoms such as loss of energy, recurrent thoughts of death and a decrease in functioning. He was treated with different medications, but there was no improvement in his mental state.

**Methods:**

For the last six months, he was treated with Esketamine and also participated in the therapy group.

**Results:**

According to content that he raised in the sessions of the therapy group and following conversations with him and with his close environment, we observed a clinical improvement in his condition – a positive mood, a decrease of the thoughts of death, an increase in his function at work and at home.

**Conclusions:**

He reported that the improvement is more significant because of the therapy group – he found a peer group, a place to process his treatment experience and to share his feelings and thoughts. We will present vignettes to demonstrate.

**Disclosure:**

No significant relationships.

